# Beyond the Psychiatric Lens: Difficulties in Diagnosing Anti-N-Methyl-D-Aspartate Receptor Encephalitis With Psychiatric Presentation

**DOI:** 10.7759/cureus.97226

**Published:** 2025-11-19

**Authors:** Madeline Bleier, Chelsea Corinaldi, Carmen Nichita, Alexander Banashkevich, Luba Leontieva

**Affiliations:** 1 Psychiatry and Behavioral Sciences, State University of New York Upstate Medical University, Syracuse, USA; 2 Radiation Oncology, State University of New York Upstate Medical University, Syracuse, USA

**Keywords:** anti-nmdar encephalitis, anti-n-methyl-d-aspartate receptor encephalitis, autoimmune encephalitis, first episode psychosis, neuropsychiatric symptoms, ovarian teratoma, young adult female

## Abstract

Anti-N-methyl-D-aspartate receptor (anti-NMDAR) encephalitis is an autoimmune disorder that predominantly affects young women and is often associated with ovarian teratomas. We describe a 21-year-old woman with a history of anxiety who initially presented with agitation, abnormal movements, and headaches. Her symptoms were first attributed to psychiatric causes, leading to multiple emergency department visits and eventual psychiatric admission. Despite escalating antipsychotic treatment, her condition rapidly worsened, marked by severe agitation, hypersexuality, and nihilistic delusions. Two weeks into her hospitalization, positive serum anti-NMDAR antibodies and pelvic imaging revealing a right ovarian teratoma prompted a shift in management to immunotherapy and surgical resection. She subsequently experienced significant cognitive recovery. This case highlights how the predominance of psychiatric symptoms in women with anti-NMDAR encephalitis can obscure timely recognition of the underlying neurologic disease. Early consideration of autoimmune encephalitis in young women with abrupt, treatment-resistant psychiatric symptoms is critical to avoid delays in diagnosis and improve outcomes.

## Introduction

Anti-N-methyl-D-aspartate receptor (anti-NMDAR) encephalitis is a disorder characterized by the production of IgG antibodies targeting the NR1 subunit of NMDA receptors, glutamate receptors critically involved in synaptic plasticity, learning, and memory [1,2].

Epidemiologically, anti-NMDAR encephalitis primarily affects people assigned female at birth, especially those in their twenties and thirties [3]. An estimated 80-100% of patients initially present with psychiatric symptoms such as agitation, delusions, hallucinations, or disorganized behavior, followed by neurologic symptoms including focal or generalized seizures, memory deficits, movement abnormalities (e.g., orofacial dyskinesias), catatonia, dysautonomia, and altered consciousness [3-5]. These symptoms often evolve in stages and may ultimately lead to life-threatening complications if not promptly addressed [4].

An estimated 30% to 60% of patients of reproductive age diagnosed with anti-NMDAR encephalitis are found to have an ovarian teratoma [3]. Therefore, in young women presenting with new-onset psychiatric and/or neurologic symptoms, a high index of suspicion should be maintained.

Early detection of this disorder is crucial, as early treatment initiation is associated with improved outcomes [3,6]. Due to the prevalence of psychiatric symptoms in the early stages of the disease, misdiagnosis often contributes to delayed treatment and worse outcomes [4,5].

Workup of suspected anti-NMDAR encephalitis requires a multidisciplinary approach. Magnetic resonance imaging (MRI) of the brain is often normal or shows nonspecific findings such as T2/FLAIR (T2-weighted-fluid-attenuated inversion recovery) hyperintensities [1,6]. Cerebrospinal fluid (CSF) analysis typically demonstrates early lymphocytic pleocytosis and later emergence of oligoclonal bands. While detection of anti-NMDAR antibodies confirms the diagnosis, autoimmune encephalitis encompasses a heterogeneous group of disorders that may involve alternative antibodies or remain seronegative [3,5,7]. Clinicians should therefore maintain a high index of suspicion and consider initiating empiric treatment when the clinical presentation is strongly suggestive. Approximately 90% of cases reveal electroencephalogram (EEG) abnormalities, most commonly diffuse slowing or, more rarely, a pattern known as extreme delta brush [8].

In patients with ovarian teratomas, treatment involves surgical resection of the tumor as a cornerstone of therapy and may also include immunotherapy such as intravenous immunoglobulin (IVIG), high-dose corticosteroids, or plasmapheresis [3,8]. The association between ovarian teratomas and anti-NMDAR encephalitis is well established. Teratomas containing neural tissue may express NR1 NMDA receptor antigens, triggering antibody formation that crosses the blood-brain barrier and binds neuronal receptors, leading to receptor loss and synaptic dysfunction [9].

In this case report, we present a 21-year-old female who presented to several different emergency departments with anxiety and migraines. After several prior ED presentations, she received workup and was ultimately diagnosed with anti-NMDAR encephalitis associated with a 3.5-cm right ovarian teratoma. This case highlights the importance of early detection through thorough history taking and physical examination, including gynecologic evaluation, when encountering atypical or rapidly progressive neuropsychiatric presentations in young female patients to improve the prognosis of this potentially life-threatening condition. Notably, this was the second recently confirmed case of anti-NMDAR encephalitis on our psychiatric unit, the first being reported by Neerukonda et al. (2020), in which an 18-year-old female presented with excited catatonia [10].

## Case presentation

A 21-year-old Caucasian woman with a psychiatric history of anxiety presented to the emergency department (ED) with her parents. The patient reported intermittent numbness on the left side of her body, headaches, and uncontrollable spasms for the past seven days. Symptoms had begun suddenly. Notably, she and her family had visited several other EDs in the preceding week, each time discharged with outpatient follow-up to her primary care provider. Prior computed tomography (CT) scans and magnetic resonance imaging (MRI) at outside hospitals were performed; however, her parents reported there were no significant findings.

On arrival, she was mildly tachycardic (heart rate 105) but otherwise normotensive, afebrile, and saturating 97% on room air. On examination, she appeared very anxious, crying, hyperventilating, and clutching her father’s hand. A urine drug screen was positive for cannabinoids. She confirmed regular cannabis use until five days prior to presentation to the ED, at which point she reportedly had discontinued use.

Initial laboratory workup revealed a comprehensive metabolic panel (CMP) that was largely unremarkable, except for low bicarbonate (15 mmol/L), low phosphorus (2.1 mg/dL), and a mild AST elevation (36 U/L, likely secondary to acetaminophen used for headaches). Complete blood count showed leukocytosis (WBC 10.3 × 10³/µL, Hgb/Hct 15.8 g/dL/46.1%). Thyroid function tests were normal aside from a slightly elevated T4 (1.99 ng/dL), which normalized by day 12. All reported laboratory values are presented with reference to institutional normal ranges (Table 1) for clarity.

**Table 1:** Reference laboratory ranges for reported values.

Laboratory Test	Normal Reference Range	Units
Bicarbonate (CO₂)	22–29	mmol/L
Phosphorus	2.5–4.5	mg/dL
Aspartate aminotransferase (AST)	10–40	U/L
White blood cell count (WBC)	4.0–10.0	×10³/µL
Hemoglobin (Hgb)	12.0–16.0	g/dL (female)
Hematocrit (Hct)	36–46	% (female)
Free thyroxine (free T4)	0.8–1.8	ng/dL
Serum creatinine (Cr)	0.6–1.1	mg/dL (female)
Creatine kinase (CK)	30–200	U/L
Erythrocyte sedimentation rate (ESR)	0–20	mm/hr
Vitamin B1 (thiamine)	70–180	nmol/L
Vitamin B6 (pyridoxine)	20–125	nmol/L
Vitamin B12 (cobalamin)	200–900	pg/mL
Temperature (afebrile)	97–99	°F
Heart rate	60–100	beats/min
Blood pressure	90–120/60–80	mmHg
Oxygen saturation	≥95	% (room air)
Anti-NMDAR antibody (serum/CSF)	Negative (<1:10 titer)	—

The initial differential diagnoses were varied between the ED team and the psychiatric consult service. Possibilities included somatization disorder, unspecified anxiety, complex migraines, functional neurological disorder (FND), drug-related side effects (cannabinoids), and, less likely, a postictal or partial seizure phenomenon. Basic organic causes such as thyroid dysfunction, drug toxicity, metabolic derangements, and infections were ruled out at this point.

Initial treatment in the ED included a migraine cocktail and a small dose of diazepam, with no improvement. Hydroxyzine provided mild symptomatic relief, but the patient continued to have episodes while in the ED described as "phasic," characterized by retching, drooling, dry heaving, and brief (10-20 seconds) periods of body contortion, hyperventilation, and shouting. It was noted by the ED that she remained fully conscious and responsive to verbal stimuli during these episodes, with no tonic-clonic activity or postictal features. She was initially discharged from the ED with hydroxyzine and instructed to follow up with outpatient psychiatric care.

Later that same day, she returned to the ED with worsening symptoms, including waxing and waning agitation and erratic behavior. She alternated between stamping her feet on the mattress, rapid breathing, verbalizing that she could not control her actions, and complaining of diffuse pain from her head to her legs. Moments later, she would resume calm, coherent conversation. Psychiatry was reconsulted and recommended continued use of hydroxyzine. However, this did not seem to improve her symptoms, and her behavior continued to escalate to the point that she required ketamine sedation. Neurology was consulted and diagnosed possible psychogenic nonepileptic seizures (PNES) and migraines, recommending no further workup for seizure activity, continued symptomatic treatment, and outpatient follow-up.

Due to her hemodynamic stability and the lack of findings suggestive of an organic etiology, the patient was eventually admitted to the inpatient psychiatric unit on the second day for continued management. On day 3 of her admission while on the psychiatric unit, neurology reevaluated her and again found no evidence warranting further workup for PNES. Psychiatry initiated fluoxetine and olanzapine for presumed unspecified anxiety disorder with panic attacks and unspecified trauma- or stressor-related disorder and severe agitation. Despite this, her behavior escalated, with episodes of screaming, shaking limbs, hypersexuality (constant masturbation), brief body contortions, and repeated statements that she could not control her body.

By day 6, risperidone was substituted for olanzapine due to lack of improvement. At this time, her parent discovered vape devices at home, creating the possibility of substance-induced psychotic disorder. Additionally, a primary psychotic disorder could not be excluded.

On day 8, her mental status further deteriorated, with nihilistic delusional statements consistent with Cotard’s syndrome, as she repeatedly stated, "I am dead." Her further deterioration and lack of improvement despite significant antipsychotic treatment in an otherwise antipsychotic-naive patient prompted further consideration of an underlying organic illness. A full psychosis organic workup was ordered, which included an autoimmune encephalitis panel and serum anti-NMDAR antibodies. An EEG and MRI were considered; however, the patient’s extreme agitation delayed imaging studies.

By day 10, antipsychotics were discontinued due to lack of efficacy and concern for neuroleptic malignant syndrome (NMS) after she developed low-grade fever (100.2°F), tachycardia, hypertension, elevated creatine kinase (CK 952 U/L), acute kidney injury (Cr 1.10 mg/dL), and worsening leukocytosis (18.9 ×10³/µL). Abdominal X-ray demonstrated increased stool burden, raising suspicion for severe constipation as a contributor to her abdominal pain and agitation. However, the absence of rigidity, diaphoresis, and continued ambulation argued against NMS. Nonetheless, she was transferred to the medicine floor for supportive care with IV fluids and further management of agitation.

**Figure 1:**
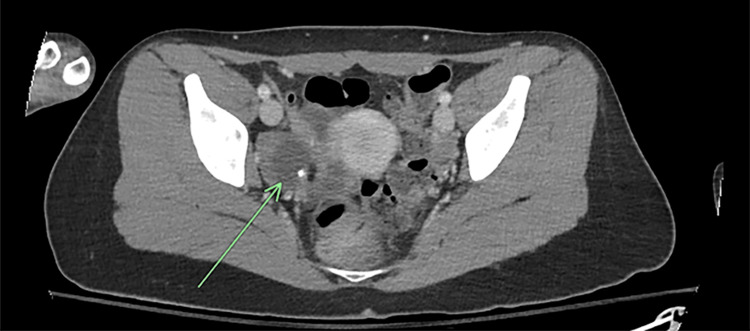
CT abdomen/pelvis, axial image. The arrow indicates 5.1 x 3.9 cm benign right adnexal teratoma.

**Figure 2:**
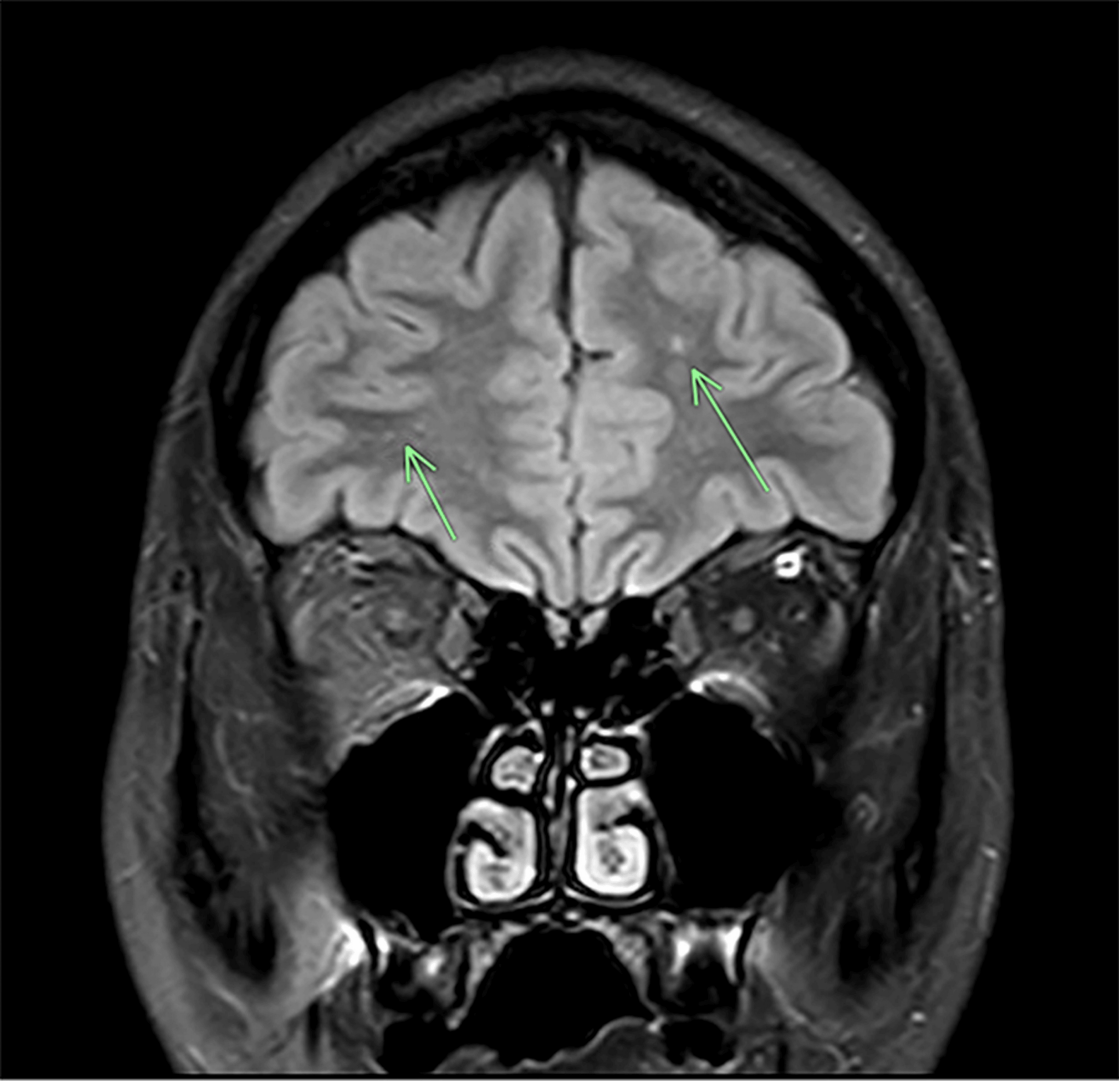
MRI brain, coronal T2-FLAIR. The arrows indicate nonspecific punctate T2-FLAIR foci in the bilateral frontal lobes. T2-FLAIR: T2-weighted fluid-attenuated inversion recovery.

On day 14, the patient’s serum anti-NMDAR antibodies returned positive, which prompted immediate consultations among the neurology, gynecology, medicine, and psychiatry teams. A pelvic ultrasound revealed a right ovarian cyst measuring 3.5 cm. Neurology recommended prompt treatment for anti-NMDAR encephalitis, initiating IV immunotherapy, steroids, and further workup from gynecology for the right ovarian cyst, in which CT scan confirmed a right ovarian teratoma (Figure 1). On day 17, the patient was placed under anesthesia for MRI of the brain and lumbar puncture (LP). The MRI indicated nonspecific punctate T2 FLAIR hyperintensities within the white matter in the bilateral frontal lobes (Figure 2). In the following days, the patient completed her five-day course of IVIG.

On day 18 of her admission, she underwent robotic-assisted right ovarian cystectomy, with pathology confirming a teratoma composed of different types of mature epithelium (skin and adnexa, columnar epithelium, minor salivary gland tissue), mature soft tissues (fat, bone, and cartilage), and brain tissue with choroid plexus, as well as focal immature cartilage and immature neural epithelium. Around day 21 of admission, results of the CSF from the LP indicated positive antibodies for NMDAR, confirming the diagnosis.

Further laboratory studies that resulted later in the hospitalization included an elevated ESR (26 mm/h on day 18), vitamin panels (B1, B6, B12) that were unremarkable apart from high B12, and negative heavy metal and porphobilinogen screening.

Following surgery and completion of IVIG, she no longer required medication for agitation. However, her mental status remained variable. Neurology recommended treatment with IV methylprednisolone, followed by an oral prednisone taper. This was slightly delayed due to concern for aspiration pneumonia, which improved with broad-spectrum IV antibiotics.

On day 28 of her admission, the patient’s cognition had finally significantly improved. She was alert, oriented, and aware of her diagnosis, though amnestic for much of her hospitalization. She expressed the desire to return to work. There was no indication for further treatment with any psychiatric medications. On day 30, she was discharged with outpatient physical and occupational therapy, a prednisone taper, and close gynecologic oncology follow-up due to the ovarian teratoma finding; no adjuvant chemotherapy was indicated.

This progressive clinical pattern, from psychiatric symptoms to motor and consciousness disturbances, is characteristic of autoimmune anti-NMDAR encephalitis. To summarize the sequence of events, Table 2 provides a chronological overview of the patient’s clinical course, diagnostic findings, and outcomes.

**Table 2:** Chronological summary of clinical course, diagnostic findings, and outcomes. ED: emergency department, NMS: neuroleptic malignant syndrome, CK: creatine kinase, NMDAR: N-methyl-D-aspartate receptor, CSF: cerebrospinal fluid, CT: computed tomography, EEG: electroencephalogram, MRI: magnetic resonance imaging, FLAIR: fluid-attenuated inversion recovery, IVIG: intravenous immunoglobulin.

Phase	Summary of Findings
Initial presentation	A 21-year-old woman with anxiety presented with seven days of progressive agitation, headaches, and abnormal movements. Noted to have predominantly psychiatric symptoms. Initial ED evaluations and imaging were unremarkable.
Psychiatric course	Admitted on day 2 for severe agitation, emotional lability, and fluctuating lucidity. No improvement with fluoxetine, olanzapine, or risperidone. By day 8, worsening neuropsychiatric symptoms and poor response to psychiatric treatment prompted evaluation for an underlying organic cause, leading to initiation of an autoimmune encephalitis workup.
Medical course	By day 10, transferred to medicine for concern of NMS (fever 100.2°F, CK 952 U/L). Serum anti-NMDAR antibody positive on day 14; CSF positive on day 21. Pelvic ultrasound on day 14 revealed a right ovarian cyst; CT abdomen/pelvis on day 16 confirmed a right adnexal teratoma.
Key diagnostics	EEG: Diffuse slowing, no epileptiform discharges. MRI: Nonspecific punctate T2/FLAIR foci. Lumbar Puncture: CSF Anti-NMDAR antibody positive; otherwise unremarkable. Pelvic Imaging: 5 cm right adnexal teratoma.
Treatment and recovery	IVIG and high-dose corticosteroids were completed by day 18; robotic cystectomy performed on the same day confirmed immature teratoma. Cognitive and behavioral recovery within two weeks; near-complete recovery by day 30 with mild short-term memory loss.
Discharge and follow-up	Discharged on day 30 with prednisone taper, outpatient physical and occupational therapy, and gynecologic oncology follow-up. No further psychiatric medication required.

While her mentation had improved, the patient was significantly deconditioned due to a long hospitalization that required prolonged periods of sedation and surgery. A long road of recovery was still ahead.

## Discussion

This case underscores the diagnostic complexity of anti-NMDAR encephalitis, particularly when psychiatric manifestations dominate the initial clinical picture. The patient’s presentation was initially consistent with primary psychiatric disorders or functional neurological symptoms, leading to multiple ED visits and eventual psychiatric admission before an organic etiology was pursued. Her early neurological symptoms, i.e., intermittent numbness, headaches, and abnormal movements, were initially overlooked due to the prominence of psychiatric features. This pattern reflects findings in the literature, where 80-100% of patients present with psychiatric symptoms, often resulting in delayed recognition and treatment [3-5]. Similar to our patient, other reported cases highlight how the early psychiatric presentation can lead to misdiagnosis as conditions such as major depressive disorder with psychotic symptoms, substance-induced psychosis, or an exacerbation of pre-existing psychiatric illnesses [10-13].

The primary reason for the delay in diagnosis was the patient’s overwhelming psychiatric presentation, which overshadowed the underlying organic etiology. Her history of anxiety and concurrent cannabis use reinforced an initial impression of a primary psychiatric or substance-related disorder. The episodic nature of her symptoms, with preserved consciousness, fluctuating agitation, and no obvious focal neurological deficits, further supported this bias. This combination of psychiatric predominance and nonspecific early workup findings prolonged the time to definitive testing for autoimmune encephalitis.

Importantly, despite treatment with antipsychotics, her clinical course declined rapidly to include severe agitation, hypersexuality, and nihilistic delusions. Failure to respond to high-dose antipsychotics should raise suspicion, as neuroleptics can exacerbate dysautonomia and movement disorders in this population.

Once the autoimmune panel was obtained, the detection of serum anti-NMDAR antibodies prompted a critical shift in management. Subsequent pelvic imaging revealed a right ovarian teratoma, in line with data showing that up to 60% of reproductive-age women with anti-NMDAR encephalitis harbor such tumors [3]. Timely initiation of immunotherapy, combined with surgical resection, represents the cornerstone of treatment and is associated with significantly improved neurologic recovery and survival [4,6].

Neuroimaging and electrophysiology provided supportive but nonspecific evidence. EEG demonstrated diffuse slowing without epileptiform activity, consistent with an encephalopathic pattern. MRI revealed nonspecific punctate T2/FLAIR foci in the bilateral frontal lobes, which, though not anatomically specific, supported cortical irritation associated with autoimmune inflammation.

Our patient’s gradual improvement after IVIG, corticosteroids, and tumor removal illustrates the reversibility of symptoms with appropriate intervention. However, her prolonged hospitalization and functional deconditioning highlight the substantial morbidity of delayed diagnosis.

This case also illustrates the critical role of interdisciplinary coordination in achieving timely diagnosis and recovery. The patient’s transition across psychiatry, medicine, neurology, and gynecology services demonstrates how fragmented care can delay recognition of autoimmune encephalitis when psychiatric symptoms dominate early presentation. Improved communication between psychiatric and medical teams, particularly regarding atypical neuropsychiatric presentations and poor antipsychotic response, may prevent such diagnostic delays. From a practical standpoint, in psychiatric settings, failure to show improvement after high-dose antipsychotic therapy should prompt evaluation for organic causes, including autoimmune encephalitis. Embedding this threshold into acute psychiatric workflows could accelerate recognition of anti-NMDAR encephalitis and improve outcomes.

Limitations

This single-case report is inherently limited in generalizability and reflects the experience of one tertiary academic medical center. Additionally, subtle documentation gaps from outside emergency departments limited the retrospective analysis of symptom evolution. Nonetheless, this case adds to a growing body of evidence illustrating the diagnostic challenges and interdepartmental coordination required for the timely identification of autoimmune encephalitis.

## Conclusions

Anti-NMDAR encephalitis exemplifies the complex intersection between psychiatry and neurology, where an autoimmune process can initially masquerade as a primary psychiatric illness. This case emphasizes the need for heightened clinical suspicion in young women with abrupt-onset neuropsychiatric symptoms, poor response to psychotropics, or fluctuating mental status. Beyond individual recognition, this case highlights a broader need for systematic education and screening protocols in acute psychiatric settings to prevent diagnostic delays. Future directions include incorporating autoimmune encephalitis awareness into psychiatric training and enhancing interdisciplinary communication. Implementing structured neurology-psychiatry consultation protocols for atypical or treatment-resistant presentations and establishing clear triggers for early autoimmune screening, such as failure to improve after high-dose antipsychotic therapy, may facilitate earlier recognition of anti-NMDAR encephalitis and reduce diagnostic delay.

In addition, consideration should be given to routine pelvic ultrasound in reproductive-age females presenting with atypical psychiatric or neurobehavioral features, as early identification of ovarian teratomas may expedite diagnosis and treatment of anti-NMDAR encephalitis. Strengthening these pathways can bridge the gap between mental and physical health disciplines, ultimately improving both recognition and recovery for patients affected by this rare but treatable condition.
